# Supplementary material for: A putative enoyl-CoA hydratase contributes to biofilm formation and the antibiotic tolerance of Achromobacter xylosoxidans
Source: NPJ Biofilms Microbiomes. 2019 Aug 6;5:20. doi: 10.1038/s41522-019-0093-6 (PMC6684605; doi:10.1038/s41522-019-0093-6)
Supplement: Supplementary file 1 — Supplemental Material [file 41522_2019_93_MOESM1_ESM.pdf]

A putative enoyl-CoA hydratase contributes to biofilm formation and the antibiotic tolerance of *Achromobacter xylosoxidans*

Cameron et al.

SUPPLEMENTARY INFORMATION

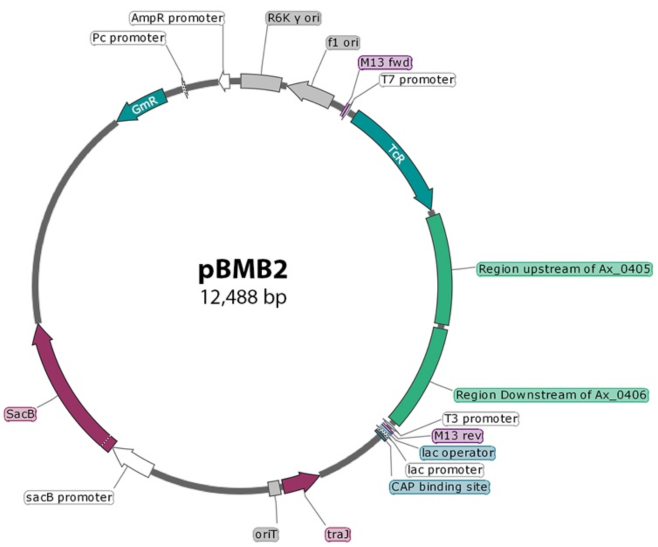

Supplementary Figure 1. Deletion and complementation constructs.

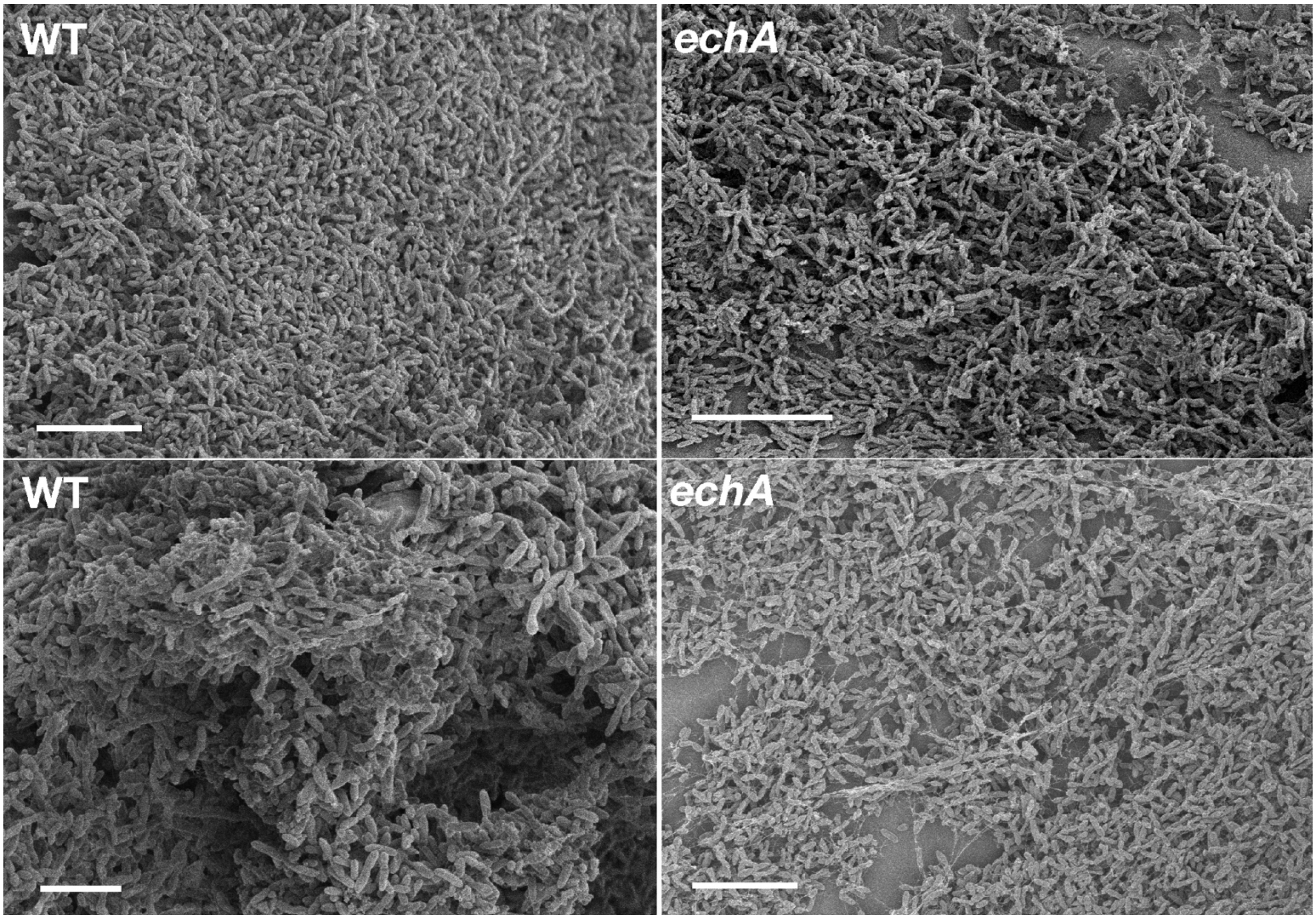

Supplementary Figure 2. Mature biofilms of the WT and *echA* mutant were visualized by SEM to examine biofilm architecture (scale bars, top = 30μm, bottom = 10 μm).

Supplementary Table 1. Strains and plasmids used in this study.

Strain or Plasmid	Characteristic	Source
A. xylosoxidans		
MN001	Cystic fibrosis clinical isolate	(1)
Δ*echA*	MN001 with a deletion of Axylo_0405 (*echA*)	This study
E. coli		
β2155	Donor strain for conjugations	(2)
WM3064	Donor strain for conjugations	(3)
UQ950	DH5α λpir	(3)
Plasmids		
pTnTet	pSC123 with Tet^R cassette from pBSL199	(4)
pSMV8	Mobilizable suicide vector, Gm^R	(3)
pBMB1	pSMV8 with Tet^R cassette from pEX18tc	This study
pEX18tc	Gene replacement vector with MCS from pUC18	(5)
pBMB2	Mobilizable suicide vector for *echA* deletion, Tet^R	This study
pBBR1MCS-5	Complementation vector	(6)
pGGA008	Cloning vector containing *alcA* promoter, Amp^R	(7)
pBMB3	Complementation vector pBBR1MCS-5 with an alcohol inducible (alcA) promoter	This study
pBMB4	Complementation vector pBBR1MCS-5::*alcAechA*	This study

Supplementary Table 2. Primers used in this study.

Primer Pair	Name	Sequence (5'-3')
1	ARB1	GGCCACGCGTCGACTAGTACNNNNNNNNNNGATAT
	TnTet1	AACAAGCCAGGGATGTAACG
2	ARB2	GGCCACGCGTCGACTAGTAC
	TnTet2	TGTCAGACCGGGGACTTATC
3	TetF	NNNGGGCCC*GCTAGCTTTAATGCGGTAGT*
	TetR	NNNNGGGCCCTGGAGTGGTGAATCCGTTAG
4	M13F	TGTAAAACGACGGCCAGT
	TetR	NNNNGGGCCCTGGAGTGGTGAATCCGTTAG
5	*echA* upF	ccctcgaggtcgacggtatcgataTTCAGGGTCAGTTCGCTCAT
	echA upR	TGTTCCAGCGTGATATCGGT
6	*echA* downF	ACCGATATCACGCTGGAACAGCTCATGCGAAGGTCCTGG
	echA downR	cggtggcggccgctctagaactagtACACGTGACGCCGTTATGC
7	*alcA*_F	cgatAAGCTTcgggatagttccgacctaggatt
	*alcA*_R	cgatGGATCCttatatagatgttcagctatgcg
8	*echA*_F	atcgGGATCCatgaccgatatcacgctgg
	*echA*_R	cgatGAGCTCtcagaagggcgagtcgg

Supplemental References

1. Badalamenti JP, Hunter RC. Complete genome sequence of *Achromobacter xylosoxidans* MN001, a cystic fibrosis airway isolate. *Genome Announc* 2015;3(4).
2. Dehio C, Meyer M. Maintenance of broad-host-range incompatibility group p and group q plasmids and transposition of tn5 in bartonella henselae following conjugal plasmid transfer from escherichia coli. *J Bacteriol* 1997;179(2):538-540.
3. Saltikov CW, Newman DK. Genetic identification of a respiratory arsenate reductase. *Proc Natl Acad Sci U S A* 2003;100(19):10983-10988.
4. Chiang SL, Rubin EJ. Construction of a mariner-based transposon for epitope-tagging and genomic targeting. *Gene* 2002;296(1-2):179-185.
5. Hoang TT, Karkhoff-Schweizer RR, Kutchma AJ, Schweizer HP. A broad-host-range flp-frt recombination system for site-specific excision of chromosomally-located dna sequences: Application for isolation of unmarked pseudomonas aeruginosa mutants. *Gene* 1998;212(1):77-86.
6. Kovach ME, Elzer PH, Hill DS, Robertson GT, Farris MA, Roop RM, Peterson KM. Four new derivatives of the broad-host-range cloning vector pbbr1mcs, carrying different antibiotic-resistance cassettes. *Gene* 1995;166(1):175-176.
7. Lampropoulos A, Sutikovic Z, Wenzl C, Maegele I, Lohmann JU, Forner J. Greengate---a novel, versatile, and efficient cloning system for plant transgenesis. *PLoS One* 2013;8(12):e83043.
